# A cross sectional study of impact and clinical risk factors of antipsychotic-induced OCD

**DOI:** 10.1016/j.euroneuro.2019.06.006

**Published:** 2019-08

**Authors:** Marjan Biria, Fiona-Xiaofei Huang, Yulia Worbe, Naomi A. Fineberg, Trevor W. Robbins, Emilio Fernandez-Egea

**Affiliations:** a Behavioural and Clinical Neuroscience Institute, University of Cambridge, UK; b Department of Psychology, University of Cambridge, UK; c Fulbourn Hospital, Cambridgeshire and Peterborough NHS Foundation Trust, Cambridge, UK; d Sorbonne Université, Paris 05, France; e Department of Neurophysiology, Saint-Antoine Hospital, Paris, France; f INSERM U 1127, CNRS UMR 7225, Institute du Cerveau et de la Moelle Epinière, Paris, France; g Hertfordshire Partnership University NHS Foundation Trust, University of Hertfordshire, Welwyn Garden City, UK; h Postgraduate Medical School, University of Hertfordshire, Hatfield, UK; i Clozapine clinic, Cambridgeshire and Peterborough NHS Foundation Trust, Cambridge, UK; j Department of Psychiatry, University of Cambridge, UK

**Keywords:** Obsessive compulsive disorder, Schizophrenia, Antipsychotic, Clozapine

## Abstract

A large proportion of schizophrenia patients treated with second generation antipsychotics will develop Obsessive Compulsive Disorder (OCD). However, there are few studies about the impact of this comorbidity and who is at higher risk. In this study of clozapine-treated patients, we aimed to determine the impact on outcome of clozapine-induced OCD, as well as the clinical and sociodemographic risk factors related to OCD-onset in clozapine patients. We had strict and novel inclusion criteria to minimise mis-identification of cases. The Obsessive-Compulsive Inventory-Revised (OCI-R) was used to divide 231 clozapine-treated patients into extreme cases of OCD (OCI  ≥ 24 or checking subscale ≥6) versus non-OCD (OCI <15 and checking subscale <4). The Global Assessment of Functioning (GAF), short version of Warwick-Edinburgh Wellbeing scale and Clinical Global Impression for schizophrenia (CGI) scales were used to determine outcome. Socio-demographic information was used to identify the risk factors for OCD development. We found that schizophrenia patients with OCD symptoms had a significantly lower patient rated wellbeing scores (*p* < 0.001) only (no difference in clinician rated wellbeing scores), higher CGI positive (*p* < 0.01) and higher CGI depressive scores (*p* < 0.05). The only risk factors that reached significance level were higher treatment dose (*p* < 0.01) and younger paternal age at birth (*p* < 0.05). There is scope for future studies based on e.g. imaging and genetic studies to further investigate causality, and in improving clinician screening for OCD.

## 1 Introduction

Schizophrenia and obsessive-compulsive disorder (OCD) are frequently comorbid (McGlashan, 1984). Several studies have shown that schizophrenia patients treated with second generation antipsychotics will develop symptoms of obsessive-compulsive disorder (OCD) (Schirmbeck and Zink, 2012) with the proportion being around half (Fernandez-Egea et al., 2018). To date, no replicated clinical risk factors have been found that help to identify vulnerable cases prior to treatment initiation. The clinical impact of this comorbidity on the patients’ social functioning and quality of life is largely unknown.

Indeed, studies investigating patient outcome and level of functioning in antipsychotic induced OCD are sparse and need further clarification (De Haan et al., 2013, Frias et al., 2014, Kim et al., 2015, Mukhopadhaya et al., 2009, Seng et al., 2018, Üçok et al., 2014). Studies have shown lower scores in self rated and foreign rated measures of wellbeing in those cases with antipsychotic-induced OCD compared to those without, with higher depressive symptoms, suicidality, hospitalisation, poorer physical and psychological health (De Haan et al., 2013, Kim et al., 2015, Seng et al., 2018, Üçok et al., 2014). In addition, interpersonal relationship scores were lower in those with OCD (Üçok et al., 2014) and OCD cases may also show more motor symptoms (Mukhopadhaya et al., 2009). Alternatively, one study showed no significant difference in outcome measures (Frias et al., 2014) when using foreign rated measures such as the Quality of Life Scale. Some studies which have investigated cognitive outcomes eg with an fMRI approach and in longitudinal studies (Schirmbeck et al., 2013, 2015) found specific and longitudinally stable cognitive impairments associated with OCD comorbid schizophrenia.

Therefore, further studies of larger and well characterised samples are needed.

There are no reliable clinical risk factors identified for developing OCD that can help the clinician to predict who will be at greater risk. Indeed, even the exact mechanism and prevalence of antipsychotic-induced OCD remain unclear. Those antipsychotics with greater anti-serotoninergic action, such as clozapine and olanzapine, have been more often associated with OCD onset (Poyurovsky et al., 2001, Schirmbeck et al., 2011). The prevalence ranges from less than 5% (Mahendran et al., 2007) to 74% (Schirmbeck et al., 2011), reflecting not only different methodologies used but also the differences in the clinical samples included. However, only a few of these studies (Fontenelle et al., 2012, Poyurovsky et al., 2005) were investigating OCD risk factors specifically in cohorts of schizophrenia patients, and they had limited sample size. Fontenelle et al. (2012) found greater severity of depression and family history of OCD in the cohort with comorbid OCD. Poyurovsky et al. (2005) also found that relatives of OCD-schizophrenia patients had higher morbid risk. Recent study also pointed to the possibility of co-prevalence of motor disorders prior to OCD development in psychotic disorders (Fineberg et al., 2017, Swets et al., 2018). Nevertheless, these findings need further replication in larger samples.

It is likely that antipsychotic-induced OCD and ‘pure’ OCD might share risk factors such as genetic, neurodevelopmental, immune and perinatal triggers. Demographic risk factors for ‘pure’ OCD include advanced maternal (Chudal et al., 2017, Steinhausen et al., 2013) and paternal age (Wu et al., 2012), perinatal events such as preterm birth, prolonged birth, low birth weight, breech, caesarean and forceps deliveries (Brander et al., 2016, Sampaio et al., 2009). Cross sectional population studies suggested that gender modulated the risk of OCD onset according to age with the highest risk in adolescent males (Steinhausen et al., 2013) and adult females (Crum and Anthony, 1993, Fontenelle and Hasler, 2008). Later in life, social factors such as social isolation, previous physical abuse (Grisham et al., 2011), maternal overprotection (Wilcox et al., 2008) and drugs, especially cocaine and cannabis (Crum and Anthony, 1993) misuse were recognised as risk factors for ‘pure’ OCD. There is a substantial body of work exploring genetic risk factors. Multiple candidate genes have been found for ‘pure’ OCD (Mattheisen et al., 2015) and associations with candidates such as the SLC1A1 gene has been found in both primary and antipsychotic associated OCD. This has however been difficult to replicate in follow up studies across ethnicities (Schirmbeck and Zink., 2013). In addition, genes have been found which share polygenic risk with both OCD and schizophrenia (Costas et al., 2016)

One potential explanation of the lack of clinical risk factors for developing OCD in antipsychotic-treated patients could be the mis-identification of cases. In a recent report by Fernandez-Egea et al. (2018) using a large cohort of clozapine treated patients, we identified a few factors that might have acted as confounders in prior studies. For instance, we found rising OCD prevalence and checking compulsion to be associated with increasing years of treatment. Development of OCD in this cohort could be a combination of underlying vulnerability and anti-psychotic treatment, so it is possible that a patient without OCD after 5 years of treatment might still be mis-identified as non-case despite going on to develop OCD later. We also found that psychosis severity correlated with OCD and obsessions severity which could potentially over-represent OCD cases in those more floridly psychotic. Such distinctions have barely been considered in prior studies of the sociodemographic and clinical risk factors of antipsychotic-induced OCD.

Our aims for this study were to a) determine if comorbid OCD is associated with a poorer outcome measures such as global functioning and subjective wellbeing, and b) identify the clinical and sociodemographic factors associated with antipsychotic-induced OCD. We used the electronic records of a large cohort of clozapine treated patients who were carefully clinically phenotyped, including using standardised scales for OCD. In order to maximise the group differences, decided on a strategy of selecting the two ends of the clinical phenotype (with and without OCD) of clozapine-treated patients as this drug is more strongly associated with OCD-onset. We then performed a whole sample analysis as a confirmatory step.

## 2 Experimental procedures

It is of note that we covered the prevalence and complex phenomenology of obsessions and compulsions in clozapine-treated patients in a previous work (Fernandez-Egea et al., 2018). Here, we use the term OCD to define these phenomena, albeit acknowledging the open debate about its pathophysiology and correct terminology.

### 2.1 Study design

This is a cross sectional, single centre study of a cohort of clozapine treated patients at the Cambridgeshire and Peterborough NHS Foundation Trust. The study included anonymised electronic clinical records of all cases from August 2015 to April 2018, embedded in an ethically approved database for research and clinical purposes (13/EE/0121). All cases were reviewed by the same care consultant (EFE).

### 2.2 Participants

The clinical records of all schizophrenia patients treated with clozapine were initially included in this study.

### 2.3 Assessment

Routine clinical assessments are described elsewhere (Fernandez-Egea et al., 2018) and include full psychiatric history, comprehensive mental state examination, current medication list, smoking habit, legal and illegal drug history, early life history, clozapine treatment length and side effects assessment and physical health assessment. Among others, psychopathological scales included assessment for general functioning [Global Assessment of Functioning (GAF)](Haro et al., 2003), short version of the Warwick-Edinburgh Wellbeing Scale [SWEWBS](Brown et al., 2016) and symptom severity using the Clinical Global Impression (CGI) for Schizophrenia (Busner and Targum, 2007) which includes 5 domains (positive, negative, cognitive, and depressive) rating from 1 to 7 for absence to extreme severity.

The Obsessive-Compulsive Inventory Revised version (OCI-R) was the questionnaire used for determining cut off scores for OCD diagnosis (Foa et al., 2002). This widely used 18 item self-rated questionnaire is done annually in these patients. Each question has a five-point Likert-type score measuring the degree of distress experienced with common OCD phenomena (not at all to extremely). It also contains six sub-scales measuring severity for obsessions, checking, washing, hoarding, neutralising and ordering. A cut-off of 21 for the total scale or above 5 for subscales is considered for OCD diagnosis (Foa et al., 2002). In a previous study, we showed that OCI-R and its six-factor component had a valid goodness-of-fit and structure in clozapine-treated patients (Fernandez-Egea et al., 2018).

### 2.4 Inclusion and exclusion criteria

The exclusion criteria were: 1) cases with no primary psychotic disorder (off label use of clozapine), 2) those cases in which documented OCD predated clozapine use and 3) and cases that neither showed clear OCD symptoms nor had a clear absence of OCD symptoms. The latter criterion was set to maximise the group differences and reduce false positives and we selected the two ends of the clinical phenotype (with and without OCD). We considered the duration of treatment, severity of the obsessive-compulsive symptoms and the checking symptoms severity, as they are the most commonly reported symptoms. Length of clozapine treatment was relevant as some might have not yet expressed the vulnerability. In this study, OCD was considered if an OCI-total score  ≥ 24 or checking subscale  ≥ 6 regardless of treatment duration. Whereas an OCI-total score <15, checking subscale <4, no treatment for OCD, and a treatment duration longer than 5 years for the non-OCD group were considered. To note, we used a more strict score criteria for OCD, of 24 instead of 21, in order to include cases with uncontroversial OCD diagnosis.

### 2.5 Statistical analysis

All statistical analyses were conducted using SPSS v23.0, with a two-tail 0.05 significance level and R studio Version 1.0.136. Data are presented as mean (*M*) and standard deviation (SD). Two independent data analyses were performed as follows: the impact of developing antipsychotic-induced OCD was assessed comparing global functioning and subjective wellbeing using a series of Multivariate ANOVAs (MANOVA). To identify the risk factors for developing OCD, we used the following list of potential risk factors for both antipsychotic-induced OCD and general OCD literature: paternal and maternal age, birth weight, hand dominance, family history of psychosis and OCD, age at presentation of schizophrenia, gender, treatment dose and duration, smoking habit, comorbidities with other psychiatric disorders and whether or not the psychosis was initially triggered by drug use. For this part of the analysis, we used 2-tailed Students t-tests for normally distributed continuous data, Mann–Whitney *U* test for not normally distributed continuous data and χ^2^ test for categorical variables and a MANOVA according to the analysis needs. A confirmatory analysis was conducted with the whole sample using a Multivariate Linear regression.

## 3 Results

The database contained two hundred thirty-one patients on clozapine with primary diagnosis of non-affective psychosis. Of those, seventy-four were missing the OCI-R scores and were not included in the analysis. Eight patients were excluded due to prior OCD diagnosis. One patient was excluded due to confirmed learning disability.

Sixty-three patients were excluded for having an OCI-R total score between 15 and 24 and/or a checking subscale score between 4 and 6. The final sample of 85 participants consists of 29 schizophrenia patients without OCD symptoms (4 females, 25 males; mean age = 45.24, SD = 12.66, range: 27–70 years) and 56 patients with OCD symptoms (14 females, 42 males; mean age 48.29 SD = 9.76, range: 29–69 years). Eleven out of 56 patients in the OCD group and 6 out of 29 patients in the non-OCD group were missing their parent's age at birth. Two participants in the non-OCD group were missing the hand dominance data. 11 patients in the non-OCD group and 24 in the OCD group did not know their birth weights. 17 participants without OCD and 31 with OCD symptoms could not provide their family history. Table 1 shows the descriptive statistics and the significance values for differences between OCD and non-OCD groups for all variables.

**Table 1:** Description of the risk factors.

	OCD	Non-OCD	*p*
*N*	56	29	
Age at presentation (*M* ± SD)	21.36 ± 4.9	21.34 ± 5.9	0.518
Gender male (%)	75	86.2	0.275
Father's age at birth (*M* ± SD)	29.6 ± 6.11	33.52 ± 8	0.028*
Mother's age at birth (*M* ± SD)	27.02 ± 5.06	30.17 ± 6.33	0.056
Treatment dose (*M* ± SD)	349.55 ± 123.35	264.66 ± 144	0.006^⁎⁎^
Treatment duration (*M* ± SD)	16 ± 7.7	16.14 ± 6.24	0.951
Low birth weight (%)	8.9^a^	6.9^a^	0.786
Current smoking (%)	32.1	31	0.56
Comorbidities (%)			0.667
Depression/schizoaffective disorder	10.7	6.9	
Anxiety/panic/phobia	5.4	10.3	
PTSD	0	3.4	
Drug/alcohol misuse	5.4	10.3	
Family history (%)			0.728
Psychosis/bipolar disorder in 1st degree relatives	12.5	24.1	
OCD/affective disorders in 1st degree relatives	14.3	10.3	
Psychosis/bipolar disorder in 2nd degree relatives	10.7	6.9	
Hand dominance (%)			0.261
Right	76.8	69	
Left	17.9	20.7	
Both	5.4	3.4^b^	
Psychosis triggered by drug use (%)	46.4	48.3	0.733
Type of presentation (%)			0.052
Hallucination/delusion	94.6	79.3	
Psychosis part of mania/affective disorder	1.8	10.3	
Non-positive presentation	0	6.9	
Unknown	3.6	3.4	

*Note: p* represents *p*-significance group differences (OCD vs non-OCD) using student's t for father's age at birth, treatment dose and duration, Mann–Whitney *U* for mother's age at birth and age at presentation, and the χ2 test for gender, low birth weight (<2500 g), current smoking, comorbidities, family history, hand dominance, psychosis triggered by drug use, and type of presentation.

a 24 out of 56 patients in the OCD group and 11 out of 29 patients in the non-OCD group did not have their birth-weight information.

b Handedness information of two patients in the non-OCD group were missing. The significance levels of *p* < 0.05 and *p* < 0.01 are indicated with one and two stars respectively.

### 3.1 Impact of antipsychotic-induced OCD in patient rated wellbeing and functioning

A series of MANOVA were conducted to assess the impact of OCD versus no-OCD (independent variable) on the GAF, CGI-Positive, CGI-Negative, CGI-Depressive, CGI-Cognitive, CGI-Overall, and the Wellbeing scores.

**Fig. 1:**
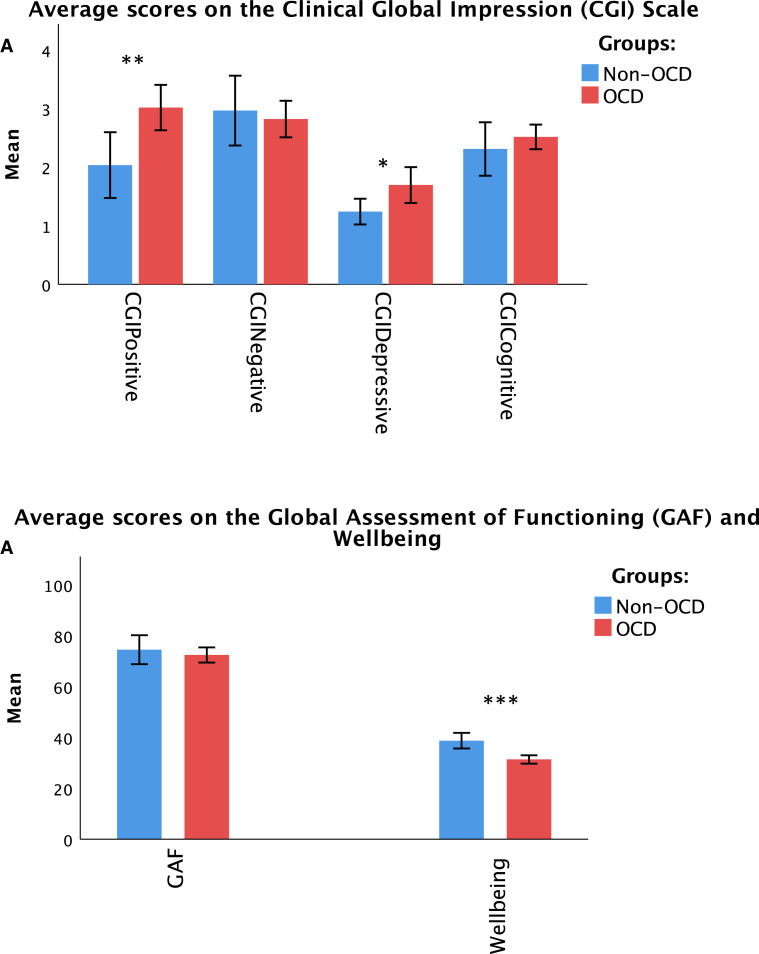
(A) Shows the average scores on different subsets of the Clinical Global Impression (CGI) scale. (B) Shows the average scores on the Global Assessment of Functioning (GAF) rated by clinicians and the Wellbeing scores rated by patients themselves. The Wellbeing scores are converted to percentages. The significance levels of *p* < 0.05, *p* < 0.01, and *p* < 0.001 are indicated with one, two and three stars respectively.

The results (Fig. 1) show a significant effect of group on wellbeing scores, *F*(1, 83) = 22.017, *p* < 0.001. Patients with OCD symptoms had a lower wellbeing score (*M* = 21.95, SD = 4.33) compared with the non-OCD group (*M* = 27.10, SD = 5.60). The presence of OCD also had a significant effect on the CGI-Positive scores, *F*(1) = 18.48, *p* < 0.01). There were higher scores in the OCD-schizophrenia group (*M* = 3.0, SD = 1.44) compared with the schizophrenia group without OCD (*M* = 2.0, SD = 1.47). In addition, the MANOVA results showed that belonging to OCD versus non-OCD group also influenced the CGI-Depressive scores significantly, *F*(1) = 4.046, *p* < 0.05, with higher depressive scores in schizophrenia-OCD group (*M* = 2.8, SD = 1.16) compared with the other group without OCD (*M* = 2.97, SD = 1.57). There was no effect of group on the GAF and the CGI-Negative scores. Table 1 shows the MANOVA results for all variables.

### 3.2 Risk factors for developing OCD

Next, we were interested to understand the clinical and sociodemographic factors associated with antipsychotic-induced OCD. A 2-tailed Students *t*-test was used to examine the impact of parents’ age, treatment dose and duration on the OCD symptoms development since these variables were continuous and normally distributed.

There were significant differences between groups for the father's age at birth and the treatment dose. Significantly more patients with OCD symptoms had a younger father at birth (*M* = 29.60, SD = 6.11) and a higher treatment dose (*M* = 349.5,SD = 123.35) compared to those without OCD symptoms who had an older father (*M* = 33.52, SD = 8) and were on a lower dose (*M* = 264.66, SD = 144), the *t*-test results are respectively *t*(66) = 2.25, *p* = 0.028 and *t*(83) = 2.84, *p* < 0.01. However, there was no significant difference between groups for the number of years they have been treated with clozapine *t*(83) = −0.062, *p* = ns.

A Mann–Whitney *U* test was performed to assess the associations between belonging to each group and the mother's age at birth and the patient's age at first presentation of psychosis since these variables were continuous and not normally distributed. The mother's age at birth was lower in the OCD group (*M* = 27.02; SD = 5.06) compared with the non-OCD schizophrenia group (*M* = 30.17; SD = 6.33). However, this difference did not reach significance, *U* = 370.5, *p* = 0.056. There was no significant difference for the age at presentation between the two groups *U* = 742, *p* = ns.

The χ^2^ test was performed to assess the differences between groups with gender, comorbidities with other psychiatric disorders, impact of a lower birth weight, family history of psychosis and OCD, hand dominance, current smoking and whether the psychosis was triggered by drug use, as these variables were categorical in nature. None of these associations reached significance (see Table 1).

A multiple linear regression was calculated as a second confirmatory analysis using the whole sample of patients with OCI total scores as dependant variable and all the risk factor variables described above. The best model with the highest adjusted-*R*^2^ was the one with years on clozapine, gender, father's age at birth, family history of psychosis and OCD, treatment dose of clozapine and smoking habit as independent variables. A significant regression equation was found (*F*(12,110) = 1.885, *p* < 0.5), with an adjusted-*R*^2^ of 0.08. Only treatment dose was a significant predictor of the OCI total score (*t* (110) = 2.949, *p* < 0.001). The family history of psychosis together with a first degree relative OCD diagnosis seem to also be associated with higher scores of the OCI total, however, it did not reach significance level (*t* (110) = 1.853, *p* = 0.06).

## 4 Discussion

In this study, we found that schizophrenia patients with clozapine-induced OCD had a lower subjective wellbeing score but not a diminished clinician-rated level of functioning. In our sample, younger paternal age and higher clozapine dose were significant risk factors for developing OCD. Thus, we identified fewer risk factors for OCD onset compared to previous studies, which could be due to our very selective patient selection process. It is of note that we set restrictive inclusion criteria that considered OCI-R total score, checking subset, and length of treatment. This strategy was adopted to minimise the mis-identification of cases. Indeed, sixty-three patients (more than a quarter of all the cases) were excluded due to having mid-range OCI-scores, who might have not developed clinical OCD due to short exposure to clozapine.

The multiple linear regression analysis in the whole sample differed in some respects. It still confirmed that role of treatment dose seems to have a significant impact on the OCI total scores. Nevertheless, we need to be cautious with this analysis, as when using the whole sample the risk of mis-identification of cases was high. For example, a mid-range OCI score could actually be a case due to not yet being on clozapine long enough to manifest, or a non-case due to a falsely high total OCI-R score from florid psychotic symptoms. In addition, there are likely multiple other factors involved in the development of OCD in schizophrenia, not least of which are genetic factors (Schirmbeck and Zink, 2013) which have not been explored in this paper.

Patients with schizophrenia who develop OCD showed worse functioning as measured by significantly lower wellbeing scores (as subjectively scored by the patients themselves). This is in concordance with previous work (De Haan et al., 2013, Seng et al., 2018, Üçok et al., 2014), which used self rated scales to assess quality of life. In our study, a clinician-scored GAF scale was also used. Notably, there was no significant difference between groups. The previous study not finding any association between OCS and outcome measures also used clinician rated quality of life measure (Frias et al., 2014). However, the clinician rated CGI scale showed increased depressive scores in the OCD cohort, which could be an indicator of the impact of the additional diagnosis on patient wellbeing or potentially due to the greater degree of positive symptoms as they also had higher CGI-positive scores. There is an indication that clinicians can be unaware of the degree to which having OCD can negatively impact the patients’ lives. The lack of awareness can therefore lead to patients not receiving adequate treatment for their OCD symptoms. The reasons for a lack of insight could be inadequate screening, of both patient's OCD symptoms and also their personal concerns. Increasing the awareness of the comorbidity as well as the routine use of screening tools might be of help to identify and treat these patients.

In the OCD group, we found higher CGI-positive scores, implying greater degree of positive symptoms of schizophrenia in the OCD cohort compared to non-OCD group. This could be consistent with a cognitive theory of the development of OCD in anti-psychotic induced schizophrenia (Fernandez-Egea et al., 2018). We have previously found that checking was the most prevalent symptom that developed after clozapine treatment. Pending of further replication, it was proposed that there may be two OCD stages in clozapine-treated psychosis. The initial goal directed checking behaviour in OCD was triggered by positive symptoms of psychosis i.e. checking due to delusional hypervigilance. Once the obsessional positive symptoms are improved, and perhaps due to the potent clozapine-induced anti-serotoninergic action, the residual checking phenomenon became part of the habit, developing a full OCD. Therefore, whether a patient has positive symptoms initially, could affect how likely they are to develop ongoing checking behaviour. Those exhibiting mainly positive schizophrenia symptoms at the beginning being more likely to have it incorporated into the checking behaviour of OCD. There was a trend for type of initial presentation, with greater proportion of schizophrenia-OCD cohort having presented with positive symptoms; however, the association did not attain statistical significance.

Younger paternal age was the only significant socio-demographic factor associated with the onset of OCD symptoms in patients with schizophrenia, which is a novel finding. It is in contrast with the only existing study that showed an increased OCD risk with increasing paternal age (Wu et al., 2012). However, this study did not study OCD development in existing schizophrenia.

The higher treatment doses of clozapine in the OCD group replicates previous findings (Baytunca et al., 2017, Fernandez-Egea et al., 2018) and remained consistent in confirmatory multilinear regression analysis. This could have various explanations such as biological sex, where males require higher doses, and smoking status, where smokers require higher doses. However, there were no significant differences in sex or smoking status (current or historical) between groups. Previous studies have shown an association between OCD severity and clozapine dose (Mukhopadhaya et al., 2009, Schirmbeck et al., 2011). When comparisons are made between the more representative plasma clozapine levels and OCD symptoms, which would remove the issues of compliance and different pharmacokinetics, the association no longer remains significant (Fernandez-Egea et al., 2018, Schirmbeck et al., 2011). The severity of positive symptoms could be a confounding factor in the relationship between clozapine dose and OCD severity, due to more severe symptoms of schizophrenia in the OCD group leading to increased requirement of clozapine.

### 4.1 Limitations

There were strengths in this study, such as the comparatively larger number of subjects available, and the use of many well validated scoring scales. However as this is a cross sectional study, we are unable to take a longitudinal view to explore causality. We were also unable to carry out genetic studies or neuro-imaging, due to the origin of the data being from the routine clinic work. These points can however be used in preparation for future studies e.g. into genetic associations which has been planned as a follow up study to explore causality. Assessing family history in our sample was also difficult, due to the information source being the patients themselves. Patients were presenting to clinic with symptoms of psychosis, so their recollections may not have been an accurate representation. This could explain why our findings did not replicate some of the previous results (Fontenelle et al., 2012, Poyurovsky et al., 2005).

### 4.2 Conclusion

In conclusion, an additional comorbidity of OCD significantly lowers the wellbeing of schizophrenia patients on subjective scales, especially those with higher positive and higher depressive symptoms. It is clear that there is also an incongruence between physician reported and patient reported quality of life results. Further research could be warranted, with increased clinician screening for OCD in reviews, and using both types of tools in screening as a goal in the clinical setting. With regards to risk factors, there were very few specific markers, either clinical or socio-demographic which could account for the development of OCD in schizophrenia. Although paternal age and clozapine dose were significant, their effects did not account for enough of the difference between groups. The possibility of a positive family history being associated with OCD development shows there is scope for future studies based on imaging and genetic studies to further closely investigate causality.

## Author disclosures

MB was supported by her studentship from the Mental Health Research UK. YW is supported by the Association Française du syndrome de Gilles de la Tourette, Foundation de recherche Medicale and Dystonia Foundation for Medical Research (USA). NF has held research or networking grants from the ECNP, UK NIHR, EU H2020, has accepted paid speaking engagements including travel and hospitality in industry supported symposia for Abbott, SunPharma, has accepted travel and hospitality expenses from the BAP, ECNP, RCPsych, CINP, receives payment from Taylor and Francis for editorial duties. TWR was supported by Wellcome Trust Senior Investigator award 104631/Z/14/Z. EF received intramural funding from CPFT/NIHR-BRC supported setting the database.

## Role of funding source

Cambridge and Peterborough Foundation Trust internal funds though NIHR-BRC for the clinical and research database only (2012-2019).

## CRediT authorship contribution statement

**Marjan Biria:** Validation, Formal analysis, Writing - original draft, Writing - review & editing. **Fiona-Xiaofei Huang:** Resources, Validation, Writing - original draft, Writing - review & editing. **Yulia Worbe:** Supervision, Validation, Writing - review & editing. **Naomi A. Fineberg:** Supervision, Validation, Writing - review & editing, Writing - review & editing. **Trevor W. Robbins:** Supervision, Validation, Writing - review & editing. **Emilio Fernandez-Egea:** Validation.
